# Day-Ahead PM_2.5_ Concentration Forecasting Using WT-VMD Based Decomposition Method and Back Propagation Neural Network Improved by Differential Evolution

**DOI:** 10.3390/ijerph14070764

**Published:** 2017-07-12

**Authors:** Deyun Wang, Yanling Liu, Hongyuan Luo, Chenqiang Yue, Sheng Cheng

**Affiliations:** 1 School of Economics and Management, China University of Geosciences, Wuhan 430074, China; yanlingliu1223@163.com (Y.L.); lhyuan2016@163.com (H.L.); yuechenqiang@sina.cn (C.Y.); chengsheng@cug.edu.cn (S.C.); 2 Mineral Resource Strategy and Policy Research Center, China University of Geosciences, Wuhan 430074, China

**Keywords:** PM_2.5_ concentration forecasting, wavelet transform, variational mode decomposition, differential evolution, back propagation neural network

## Abstract

Accurate PM_2.5_ concentration forecasting is crucial for protecting public health and atmospheric environment. However, the intermittent and unstable nature of PM_2.5_ concentration series makes its forecasting become a very difficult task. In order to improve the forecast accuracy of PM_2.5_ concentration, this paper proposes a hybrid model based on wavelet transform (WT), variational mode decomposition (VMD) and back propagation (BP) neural network optimized by differential evolution (DE) algorithm. Firstly, WT is employed to disassemble the PM_2.5_ concentration series into a number of subsets with different frequencies. Secondly, VMD is applied to decompose each subset into a set of variational modes (VMs). Thirdly, DE-BP model is utilized to forecast all the VMs. Fourthly, the forecast value of each subset is obtained through aggregating the forecast results of all the VMs obtained from VMD decomposition of this subset. Finally, the final forecast series of PM_2.5_ concentration is obtained by adding up the forecast values of all subsets. Two PM_2.5_ concentration series collected from Wuhan and Tianjin, respectively, located in China are used to test the effectiveness of the proposed model. The results demonstrate that the proposed model outperforms all the other considered models in this paper.

## 1. Introduction

Over the past few decades, with the rapid development of industrialization and urbanization, the occurrence of haze pollution episodes has become more frequent and more severe in China [1,2]. According to the statistics of China’s National Development and Reform Commission, since early 2013, many areas including the north China, Huanghuai, Jianghuai, Jianghan, south of the Yangtze River and the north of southern China have suffered severe and continuous haze weather. Haze pollution brings serious adverse effects on the environment, clime, ecological systems, economy and public health, thus causes great harm to the human production and life on a global scale [3,4]. Even though the mechanism of haze formation is still not clear [5], the high level concentrations of fine particles with aerodynamic diameter of 2.5 μm or less (PM_2.5_) was inferred as the main reason of haze pollution episodes, and thus attracted widespread public concerns [6,7]. Compared to the PM_10_ (particulate matter with aerodynamic diameter below 10 μm), PM_2.5_ has smaller diameter and stronger adsorption capacity of hazardous materials such as heavy metal and organic pollutants, and therefore has more serious adverse effects on human health and atmosphere quality [8]. With long-term exposure to PM_2.5_, the incidence of associated diseases such as lung diseases, heart diseases and premature death in human beings will be significantly increased [9,10]. The severity of adverse effects caused by PM_2.5_ makes it an urgent need for researchers to simulate and forecast its concentration. However, due to the diverse sources, strong regional and long-range transport, multiple influencing factors, along with complex formation mechanisms, the previous studies have illustrated the difficulty in accurate prediction of PM_2.5_ concentration [11,12].

In the previous studies, many methods and tools have been proposed for predicting the concentration of PM_2.5_ or other air pollutants. These methods can be generally divided into the following three categories: (1) deterministic methods; (2) causal relationships forecasting methods; and (3) time series based forecasting methods. Deterministic methods such as community multi-scale air quality (CMAQ) model can simulate the process of discharge, accumulation, diffusion and transfer of a pollutant by employing meteorological, emission and chemistry models, and are often used for predicting the concentrations of air pollutants [13]. For instance, Djalalova et al. proposed a new post-processing method based on the CMAQ model for surface PM_2.5_ predictions [14]. However, the scale and quality of the emission data adopted make great influence on the forecast accuracy of deterministic methods [15]. Moreover, the deterministic methods need much computational time to accomplish the forecasting process [16]. Therefore, many researchers integrated the deterministic methods with intelligent or statistical methods in order to enhance the forecast accuracy. For instance, Konovalov et al. proposed a hybrid model for PM_10_ concentration forecasting based on the deterministic model and statistical model, where the statistical model was utilized to correct predictions made by the deterministic model [17]. Song et al. developed an adaptive neuro-fuzzy model to implement deterministic forecasting of PM based on the data of hourly and 12 h averaged air pollutants within Yangtze River Delta region of China [18]. Causal relationships forecasting methods work on the causal relationships between independent variables and forecast values. For instance, Sun and Sun developed a novel hybrid model based on principal component analysis (PCA) and least squares support vector machine (LSSVM) optimized by cuckoo search (CS) for daily PM_2.5_ concentration prediction. In their proposed model, the PCA is firstly adopted to obtain the most important influencing factors of PM_2.5_ concentration, then based on the selected influencing factors, the PM_2.5_ concentration is predicted using CS-LSSVM model [19]. However, as mentioned before, since the PM_2.5_ concentration is influenced by many complicated factors such as temperature, wind speed, precipitation which are very difficult to be determined accurately and quantificationally in real-world applications, thus, it is really a big challenge to build an accurate forecasting model using causal relationships forecasting method. Therefore, many researchers turn to forecasting PM_2.5_ concentration based upon the time series forecasting methods which rely on the historical series. 

The most frequently used time series forecasting methods in this study area can be further divided into the following two categories: statistical models and hybrid models. Statistical models are easy, quick and efficient, and mainly involve autoregressive integrated moving average model (ARIMA), multiple linear regression (MLR), generalized autoregressive conditional heteroskedasticity (GARCH), grey model (GM), Markov models and artificial intelligence (AI) based models. For example, Jian et al. utilized ARIMA model to investigate the effect of meteorological factors on submicron particle concentrations under busy traffic conditions [20]. Stadlober et al. developed a MLR model combining information of the present day with meteorological forecasts of the next day to forecast daily PM_10_ concentrations for sites located in Bolzano, Klagenfurt and Graz [21]. Kumar and Ridder developed a hybrid model integrated GARCH modeling technique and FFT-ARIMA to forecast daily maximum O_3_ concentration, and to make probabilistic forecasts of ozone episodes at four urban sites of two major European cities (London and Brussels); the results revealed the good forecasting performance of the proposed model [22]. Pai et al. proposed seven types of first-order and one-variable grey differential equation model to predict hourly PM concentrations in Banciao city of Taiwan, the results showed that GM(1,1) is an efficient early-warning tool for providing PM information to the inhabitants [23]. Sun et al. utilized hidden Markov models (HMMs) to forecast daily average PM_2.5_ concentration, the comparisons between different distributions used in HMMs showed that the closer the distribution employed in HMMs is to the observation sequence, the better the model prediction performance [24]. Ordieres et al. compared the forecasting capability of three different neural networks for PM_2.5_ concentration forecasting, including multilayer perceptron (MLP), radial basis function (RBF) and square multilayer perceptron (SMLP) [25]. Voukantsis et al. constructed an ANN (multi-layer perceptron) model to accomplish the forecasting of daily PM_10_ and PM_2.5_ concentrations [26].

In order to improve the forecast accuracy, hybrid models which combine some single models have been widely used in air quality forecasting field. For example, Lin et al. forecasted concentrations of air pollutants including PM_10_, NO_x_ and NO_2_ by logarithm SVM with immune algorithms (IA) [27]. Perez developed a combination model of ANN and a nearest neighbor method to conduct PM_10_ forecasting [28]. Antanasijević et al. applied the artificial intelligence model combining the genetic algorithm (GA) and ANN to forecast PM_10_ emission [29]. However, the multiple frequency components existed in PM_2.5_ concentration series are always the challenging parts in forecasting, making the models which work on the original time series cannot handle them appropriately. Thus, many decomposition techniques such as WT, EMD, VMD and their variants have been applied for dealing with the multiple frequency components before forecasting. For instance, Bai et al. utilized wavelet transform (WT) technique and back propagation neural network (BPNN) to forecast daily air pollutants (PM_10_, SO_2_ and NO_2_) concentrations, and experiment results showed that the WT-BPNN model has better forecasting performance than BPNN model in terms of the forecast accuracy [30]. Zhou et al. developed a hybrid model based on EEMD and a general regression neural network (GRNN) to predict PM_2.5_ concentration, and obtained accurate results [2]. Liu et al. presented four different hybrid models by combining four signal decomposing algorithms (e.g., wavelet decomposition (WD)/wavelet packet decomposition (WPD)/EMD/fast ensemble empirical mode decomposition (FEEMD)) and ELM model to complete the multi-step-ahead wind speed forecasting, and the experiments indicated that all the proposed hybrid models have better performance than the single ELM model [31]. Wang et al. [32] proposed a hybrid model based on two-layer decomposition method and BP neural network optimized by firefly algorithm for multi-step electricity price forecasting, and the experimental results illustrated the superior performance of the proposed model.

Although the hybrid models integrated single decomposition techniques can improve the forecasting ability to some extent, while since all the single decomposition techniques have the drawback of mode mixing with different levels, which makes the multiple frequency components existed in the PM_2.5_ concentration series cannot be effectively extracted, and consequently leads to an inferior forecasting performance. Therefore, this paper proposes a novel hybrid model based on WT-VMD decomposition method and BP neural network optimized by DE algorithm for one day ahead PM_2.5_ concentration forecasting. Firstly, WT is employed to decompose the PM_2.5_ concentration series into a number of subsets with different frequencies. Since the problem of frequency aliasing exists between the subsets may increase the forecasting difficulty, therefore, VMD is further applied to conduct the secondary decomposition of each subset generated by WT, and a set of modes are obtained. Next, BP model optimized by DE is utilized to forecast the modes generated by VMD. Finally, the forecast value of each subset is obtained through aggregating the forecast values of all modes generated from this subset by VMD, and the forecast series of PM_2.5_ concentration is obtained by adding up the forecast values of all subsets. The proposed model is tested using two PM_2.5_ concentration series collected respectively from Wuhan and Tianjin located in China. The results demonstrate that the proposed model outperforms all the other considered models in this paper.

The rest of this paper is organized as follows. Section 2 describes in detail the methodology adopted in the paper. Section 3 develops the proposed hybrid model. In Section 4, two PM_2.5_ concentration series collected from Wuhan and Tianjin, respectively, located in China are taken for conducting the empirical study. Section 4 is the concluding remarks.

## 2. Methodology

### 2.1. Wavelet Transform (WT)

**Figure 1:**
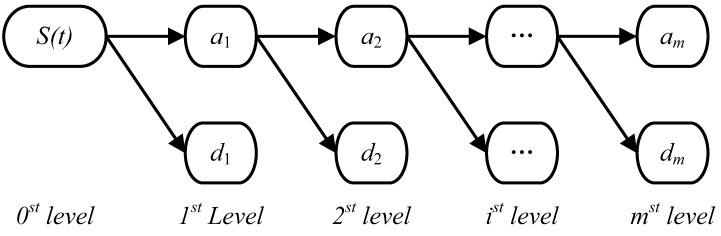
The process of m-level decomposition by WT for time series S(t).

WT is a powerful technique for processing the non-periodic, non-stationary and transient signals [33]. WT decomposes a time series into different components at different frequency levels: one low frequency approximation subset which shows the general trend of the signal and several high frequency detail subsets which are related to the noise and disturbance [34]. As shown in Figure 1, the process of m-level decomposition by WT for time series S(t) can be defined as follows:

(1) $$S(t)=d_{1}+d_{2}+\cdots +d_{m}+a_{m}$$

Compared to the original signal, these subsets generated by WT usually have some better behaviors such as more stable variance and fewer outliers, which facilitates the prediction task and therefore improves the overall prediction accuracy [35]. WTs can be divided into the following two categories: (1) continuous wavelet transform (CWT); and (2) discrete wavelet transform (DWT). 

The CWT of a signal f(t) is defined as follows:

(2) $$CWT_{f}\left(a,b\right)=\frac{1}{\sqrt{\left|a\right|}}\int_{-\infty }^{+\infty }f\left(t\right)\psi^{*}\left(\frac{t-b}{a}\right)d_{t}$$

where a and b are the parameters of scale and translation, respectively; ${}^{*}$ represents the complex conjugate; and the mother wavelet ψ(t) denotes the transforming function.

The DWT of a signal f(t) is defined as follows:

(3) $$\mathrm{DW}T_{f}\left(m,n\right)=2^{-\frac{m}{2}}\sum_{t=0}^{T-1}f\left(t\right)\psi^{*}\left(\frac{t-n2^{m}}{2^{m}}\right)$$

where the integer m is the scale factor (decomposition level), the integer n is the sampling time, T is the length of signal f(t), and t is the discrete time index.

The number of decomposition levels and selection of the mother wavelet have considerable effects on the characteristics of subsets and thus influence significantly the overall prediction error. In the decomposition process of WT, more levels will result in more stationary subsets; however, large number of levels might cause decomposition information loss and thus low prediction accuracy [34]. Based on the above considerations, this paper adopts a three-level DWT with mother wavelet of the Daubechies wavelet of order 4 (Db4), which has the ability of providing a balance between wavelength and smoothness [35].

### 2.2. Variational Mode Decomposition (VMD)

VMD is an effective signal decomposition method proposed by Dragomiretskiy and Zosso in 2014 [36]. VMD can decompose a real valued signal into a discrete set of band-limited modes (denoted by $y_{k}$) which have specific sparsity properties when producing main signal. It is assumed that each mode $y_{k}$ generated by VMD can be compressed around a center pulsation $\omega_{k}$ which is determined along with the decomposition process. In order to obtain the bandwidth of each mode, the following procedures should be accomplished: (1) for each mode $\mu_{k}$, compute the associated analytic signal with the benefit of Hilbert transform to obtain a unilateral frequency spectrum; (2) mix with an exponential tuned to the respective estimated center frequency in order to shift the mode’s frequency spectrum to baseband; and (3) estimate the bandwidth of each model through Gaussian smoothness of the demodulated signal. Then, the constrained variational problem can be provided as follows:

(4) $$\min_{\mu_{k},\omega_{k}}=\left\{{\sum_{k}\left\|\partial_{t}\left[\left(\delta (t)+\frac{j}{\pi t}\right)*\mu_{k}(t)\right]e^{-j\omega_{k}t}\right\|}_{2}\right\}$$

Subject to

(5) $$\sum_{k}\mu_{k}=f$$

where f is the original signal, μ is its mode, ω is the frequency, δ is the Dirac distribution, t is time script, k is the number of modes, and ∗ denotes convolution. Recall that, in the VMD framework, the original signal f is decomposed into a set of modes denoted μ (see Equation (5)) each having a bandwidth in Fourier domain (see Equation (4)) and compacted around a center pulsation $\omega_{k}$. The solution to the original minimization problem (see Equation (4)) is the saddle point of the following augmented Lagrangian (L) expression:

(6) $$L(\mu_{k},\omega_{k},\lambda )=\alpha {\sum_{k}\left\|\partial_{t}\left[\delta (t)+\frac{j}{\pi t}*\mu_{k}(t)\right]\right\|}_{2}^{2}+{\left\|f-\sum \mu_{k}\right\|}_{2}^{2}+\langle \lambda ,f-\sum \mu_{k}\rangle$$

where λ is the Lagrange multiplier and α represents the balancing parameter of the data-fidelity constraint. Consequently, the solutions for u and ω can be obtained based on the following two equations:

(7) $$\mu_{n}^{n+1}=\left(f-\sum_{i\neq k}\mu_{i}+\frac{\lambda }{2}\right)\frac{1}{1+2\alpha {\left(\omega -\omega_{k}\right)}^{2}}$$

(8) $$\omega_{n}^{n+1}=\frac{\int_{0}^{\infty }\omega {\left|\mu_{k}(\omega )\right|}^{2}d\omega }{\int_{0}^{\infty }{\left|\mu_{k}(\omega )\right|}^{2}d\omega }$$

where n is the number of iterations.

### 2.3. The DE-BP Model

#### 2.3.1. Back Propagation (BP) Neural Network

**Figure 2:**
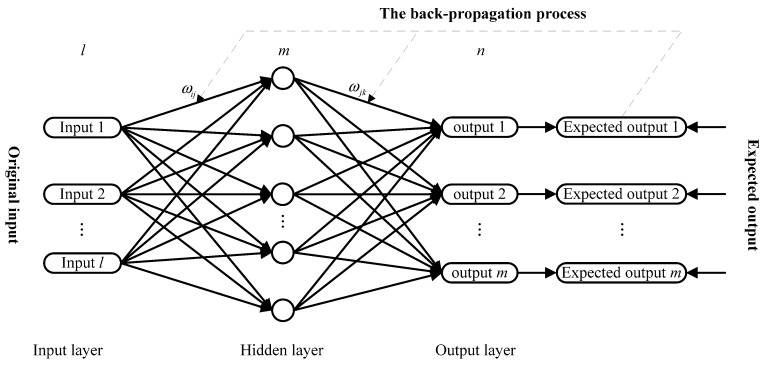
The basic structure of a three-layer BP neural network.

Artificial neural networks (ANNs) include a family of intelligent models that mimic the biological neural networks. The BP neural network including one or more hidden layers is one of the ANN models, which has a relative simple structure and thus can be realized easily. Since the distinguish performance of the BP neural network, it has been popularly used in many practical fields such as wind speed forecasting [37], plastic injection molding [38], natural gas load forecasting [39] and so forth. The BP neural network used in this study has a three-layer network consisting of an input layer, a hidden layer, and an output layer (see Figure 2). The BP neural network distinguishes itself by the presence of hidden layers whose computation nodes are correspondingly called hidden neurons. The function of hidden neurons is to connect the input and the network output. Given a training set of input-output data, the most common learning rule for multi-layer perceptron (MLP) neural networks is the back-propagation algorithm which involves two following phases: the first one is a feed-forward phase in which the external input information at the input nodes is propagated forward to compute the output information signal at the output unit; the second one is a backward phase in which modifications to the connection weights are made based on the differences between the computed and observed information signals at the output units. In this study, a tangent sigmoid function is used as the neuron transfer function.

After determination of the network topology and initialization of the associated network parameters, the BP neural network should be trained and tested through the following three steps:

**Step** **1:** Calculate the output of the *j*th node in the hidden layer using the following equation.
  (9) $$H_{j}=f(\sum_{i=1}^{n}\omega_{ij}x_{i}-a_{j})$$
  where i is the index of neuron in the input layer, n is the number of neurons in the input layer, $\omega_{ij}$ is the connection weights between input layer and hidden layer, $x_{i}$ is the *i*th input value, $a_{j}$ is threshold value, and $H_{j}$ and f represent the output of hidden layer and the incentive function of neurons, respectively.
**Step** **2:** Calculate the fitted value or forecasting value of the *k*th node in the output layer using the following equation.
  (10) $$O_{k}=\sum_{j=1}^{l}H_{j}\omega_{jk}-b_{k}\;k=1,2,\ldots ,m$$
  where $\omega_{jk}$ is the connection weights between the output layer and hidden layer, $b_{k}$ is threshold value, and m is the number of neurons in the output layer.
**Step** **3:** Calculate the fitting error $e_{k}$ based on the fitted value and expected output, and update the weight factor and threshold value by the following formula.
  (11) $$\omega_{ij}=\omega_{ij}+\eta H_{j}(1-H_{j})x(i)\sum_{k=1}^{m}\omega_{jk}e_{k}\;i=1,2,\ldots ,n;\;j=1,2,\ldots ,l$$
  (12) $$\omega_{jk}=\omega_{jk}+\eta H_{j}e_{k}\;j=1,2,\ldots ,l;\;k=1,2,\ldots ,m$$
  (13) $$a_{j}=a_{j}+\eta H_{j}\left(1-H_{j}\right)x\left(i\right)\sum_{k=1}^{m}\omega_{jk}e_{k}\;j=1,2,\ldots ,l$$
  (14) $$b_{k}=b_{k}+e_{k}\;k=1,2,\ldots ,m$$
  where η denotes the learning rate, x(i) is the *i*th input value.

The training process of the BP neural network is stopped when one of the following two conditions is satisfied: (1) the maximum number of iterations is reached; and (2) the fitting accuracy meets the requirement.

#### 2.3.2. Differential Evolution (DE) Algorithm

**Figure 3:**
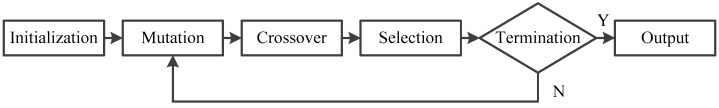
The flowchart of DE algorithm.

DE algorithm proposed by Storn and Price in 1997 is a stochastic, population-based and direct search algorithm which has the characteristics of simple structure, less control parameters, fast convergence, and strong robustness, and therefore has significant advantages for dealing with the non differentiable, nonlinear and multimodal functions [40]. As shown in Figure 3, the standard DE algorithm consists of four main operations: initialization, mutation, crossover and selection.

The basic steps of DE algorithm are illustrated as follows:

**Step** **1:** Population initialization. Initializing population of DE algorithm based on the following formula.
  (15) $$x_{j,i,o}=x_{j,\min}+rand_{i,j}\left(0,1\right)\times \left(x_{j,\max}-x_{j,\min}\right)$$
  where $x_{j,i,o}$ denotes the value of *i*th individual in the *0*th generation and *j*th dimension.
**Step** **2:** Mutation. Based on the randomly selected three indices,  m, i and  j,  m≠i≠j, a mutant vector $V_{k,G}$ is generated based on the following formula.
  (16) $$V_{k,G}=X_{m,G}+F(X_{i,G}-X_{j,G})$$
  where  k≠m≠i≠j, F is a scaling factor and F∈[0,2], $X_{m,G}$ is the base vector.
**Step** **3:** Crossover. Crossover operation is introduced into DE algorithm in order to improve the multiplicity of the perturbed parameter vectors. The trial point $U_{j,k,G+1}$ is established from its parents $V_{j,k,G+1}$ and $X_{j,k,G}$ by the following formula.
  (17) $$U_{j,k,G+1}=\left\{ \begin{array}{l} V_{j,k,G+1}\;\mathrm{if}\;(rand\;b(j)\leq C_{R})\;\mathrm{or}\;j=rnbr(i)\\ X_{j,k,G}\;\mathrm{if}\;(rand\;b(j)>C_{R}\;\mathrm{and}\;j\neq rnbr(i)) \end{array}\right.\;j=1,\ldots ,D,$$
  where $C_{R}$ is crossover probability and $C_{R}\in \left[0,1\right]$, rnbr(i) is a randomly selected index in the set of {1,2,3,…,D}, which ensures that $U_{j,k,G+1}$ obtains at least one parameter from $V_{j,k,G+1}$. The trial vector is formed of both current parameter vectors and mutant vector parameters (see formula (17)).
**Step** **4:** Selection. The trial vector $X_{i,G+1}$ can be obtained by comparing the fitness value of the vector obtained through mutation and crossover, and the process can be denoted as follows:
  (18) $$X_{i,G+1}=\left\{ \begin{array}{l} U_{i,G}\;if\;f\left(U_{i,G}\right)\leq f\left(X_{i,G}\right)\\ X_{i,G}\;otherwise \end{array}\right.$$
**Step** **5:** Iterative computing and stop the DE algorithm if the result satisfies the error requirement or the maximum number of iterations is reached. Otherwise, return to Step 2.

#### 2.3.3. The DE-BP Model

**Figure 4:**
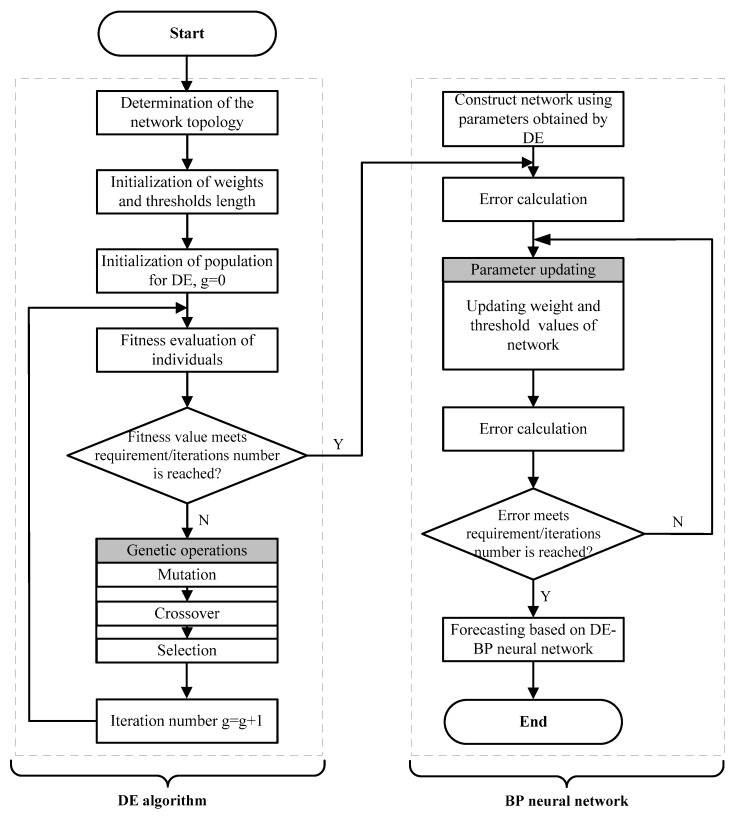
The flowchart of DE-BP model.

In the BP neural network, the two kinds of training parameters of weight matrices ($\omega_{ij}$ and $\omega_{jk}$) and thresholds ($a_{j}$ and $b_{k}$) have significant influences on the prediction accuracy. In order to improve the function approximation ability of the BP neural network, especially on the catastrophe points, in this study, DE algorithm is utilized to optimize the weight matrices and thresholds, see Figure 4. The fitness function of DE algorithm used in this study is the RMSE of forecast results, and is defined as follows:

(19) $$F_{fitness}=\sqrt{\frac{1}{N}{\sum_{t=1}^{N}\left(\hat{X}\left(t\right)-X\left(t\right)\right)}^{2}}$$

where $\hat{X}\left(t\right)$ denotes the forecast value at time t, X(t) represents the actual value at time t, and N is the total number of data. The individual owning the minimal fitness value is the global best point, which can be used to determine the parameters of the BP neural network.

The steps of DE-BP model are described as follows:

**Step** **1:** Initialization. Determine the network topology of the network and initialize the parameters of DE algorithm including population size, maximum iteration number, probabilities of mutation and crossover operators. The initial population is generated using Equation (15).
**Step** **2:** Calculate the fitness value of each individual using Equation (19). The DE algorithm is stopped when the stop criterion is satisfied, and go to Step 4.
**Step** **3:** Update the population of DE algorithm based on mutation, crossover and selection operators. Go to Step 2.
**Step** **4:** The optimal individual obtained from DE algorithm is adopted as the initial connection weights and thresholds of the BP neural network.
**Step** **5:** Train and test the BP neural network based on the training and testing samples.

#### 2.3.4. Hybrid WT-VMD-DE-BP Forecasting Model

**Figure 5:**
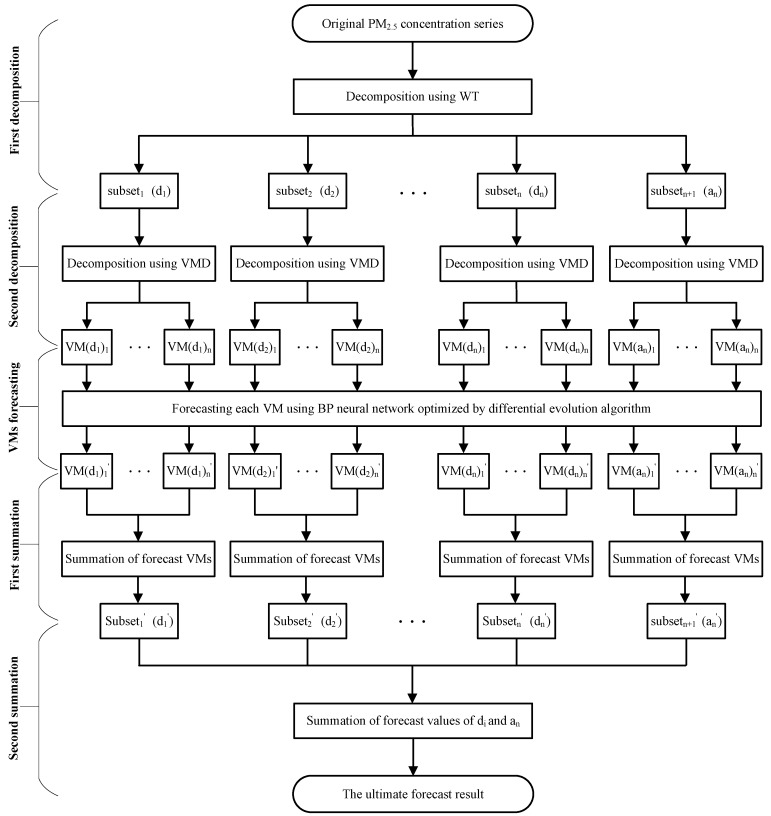
The flowchart of WT-VMD-DE-BP model.

In this section, the proposed WT-VMD-DE-BP model is established for daily PM_2.5_ concentration forecasting. As shown in Figure 5, the basic structure of the hybrid forecasting method includes the following five steps:

**Step** **1:** First decomposition. The WT decomposition technique is utilized to decompose the PM_2.5_ concentration series into one low frequency approximation subset and several high frequency detail subsets.
**Step** **2:** Second decomposition. In order to increase the forecasting accuracy, the VMD technique is further employed to conduct the secondary decomposition of each subset generated by WT, and consequently a number of VMs are obtained.
**Step** **3:** Individual forecasting. Each VM generated by VMD is forecasted using DE-BP model.
**Step** **4:** First summation. The forecast value of each subset generated by WT is obtained by adding up all the forecast values of VMs generated by VMD decomposition of this subset.
**Step** **5:** Second summation. The forecast series of PM_2.5_ concentration is obtained by aggregating the forecast result of each subset.

## 3. Empirical Study

### 3.1. Study Area and Data Description

**Figure 6:**
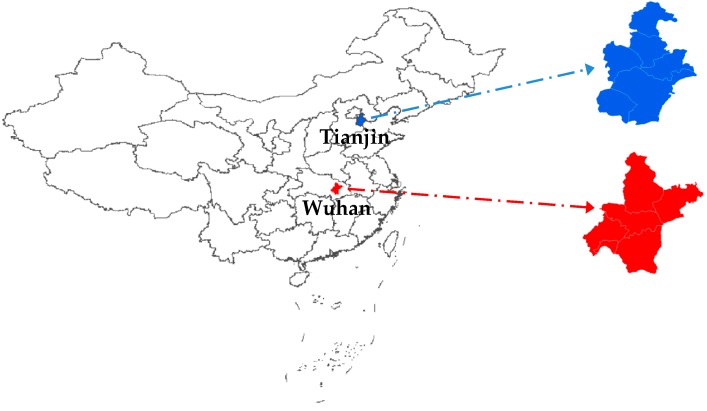
Geographical locations of the study areas.

In this paper, two PM_2.5_ concentration series respectively collected from Wuhan and Tianjin located in China are adopted for testing the validity of the proposed model. The specific locations of the two study areas are briefly depicted in Figure 6. Wuhan, situated in the middle-lower Yangtze Plain and the eastern part of Jianghan Plain (30° N and 114° E), has been regarded as China’s important industrial base, integrated transportation hub, and science and education base. The Yangtze River, which is the third longest river in the world, and the largest tributary of the Han River meet at this city, making Wuhan become a very important inland river port. Wuhan has a sub-tropical monsoon humid climate with abundant rainfall, abundant sunshine and four distinct seasons. Tianjin, the largest coastal city in northern China, is located along the west coast of the Bohai Gulf (39° N and 117° E). Tianjin has become a new growth pole and a hub of advanced industry and financial activity in China. Tianjin has a sub-humid warm temperate monsoon climate that characterized by significant winds and four distinct seasons. With rapid development of urbanization in the past several decades, both Wuhan and Tianjin become two megalopolises with a population of more than ten million people. Simultaneously, because of the development of industrialization and increase of motor vehicles, the occurrence of haze weather in these two megalopolises becomes more frequent and more severe, which makes it an urgent need for researchers and relevant government departments to simulate and forecast the PM_2.5_ concentration in order to protect public health and atmospheric environment.

**Figure 7:**
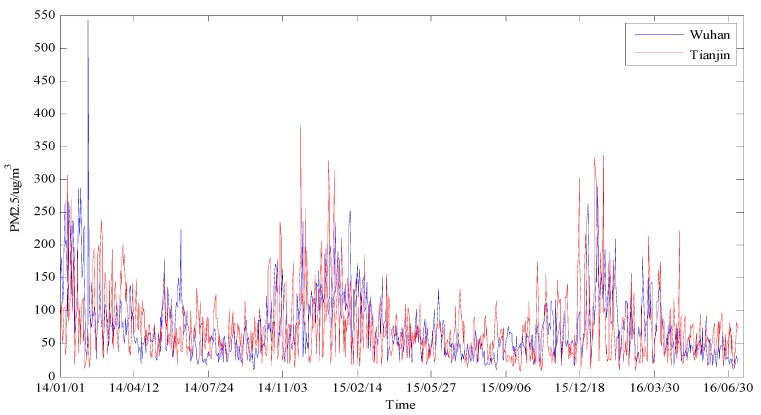
The original PM_2.5_ concentration series of Wuhan and Tianjin.

In this paper, the two original daily PM_2.5_ concentration series from 1 January 2014 to 30 June 2016 with a total of 912 observations in Wuhan and Tianjin are collected from China’s online air quality monitoring and analysis platform (http://www.aqistudy.cn/), as shown in Figure 7. In Figure 7, it can be seen that the two PM_2.5_ concentration series share some common features, for example, both Wuhan and Tianjin have high level of PM_2.5_ concentration in winter (roughly between November and February of each year). However, since Wuhan and Tianjin have different geographical positions, climatic characteristics and industrial structures, the PM_2.5_ concentration series associated with the two megalopolises appear to be different, and therefore are suitable for testing the effectiveness and practicability of the proposed forecasting model. Specifically, in each PM_2.5_ concentration series, the 1st–882nd data (1 January 2014–31 May 2016) and 883rd–912th data (1 June 2016–30 June 2016) are adopted as the training and testing samples, respectively. This study selects four accuracy measures presented in Section 3.2 in order to evaluate the performance of the proposed forecasting model. In addition, it should be noted that all considered models adopted in this paper are coded in MATLAB R2010a.

### 3.2. Performance Criteria of Forecasting Accuracy

This study adopts the following four error metrics to testify the effectiveness and practicability of the proposed forecasting model: mean absolute error (MAE), root mean square error (RMSE), mean absolute percentage error (MAPE) and Theil’s inequality coefficient (TIC). The performance measures of MAE, RMSE and MAPE are utilized to quantify the errors of forecast values, and the smaller they are, the better the prediction accuracy is. TIC is employed to evaluate the predictive capability of different forecasting models, and the smaller it is, the better the forecasting capability that the model has.

The computational formulas of these four performance measures are provided as follows:

(20) $$MAE=\frac{1}{n}\sum_{t=1}^{n}\left|\hat{x}\left(t\right)-x\left(t\right)\right|$$

(21) $$RMSE=\sqrt{\frac{1}{n}\sum_{t=1}^{n}{\left(\hat{x}\left(t\right)-x\left(t\right)\right)}^{2}}$$

(22) $$MAPE=\frac{1}{n}\sum_{t=1}^{n}\left|\frac{\hat{x}\left(t\right)-x\left(t\right)}{x\left(t\right)}\right|$$

(23) $$TIC=\frac{\sqrt{\frac{1}{n}\sum_{t=1}^{n}{\left(\hat{x}\left(t\right)-x\left(t\right)\right)}^{2}}}{\sqrt{\frac{1}{n}\sum_{t=1}^{n}x{\left(t\right)}^{2}}+\sqrt{\frac{1}{n}\sum_{t=1}^{n}\hat{x}{\left(t\right)}^{2}}}$$

where n is the number of observed PM_2.5_ concentration values; and $\hat{x}\left(t\right)$ and x(t) are the forecast and observed values of PM_2.5_ concentration at time t, respectively.

### 3.3. PM_2.5_ Concentration Forecasting in Wuhan

#### 3.3.1. Analysis of Decomposition Results

**Figure 8:**
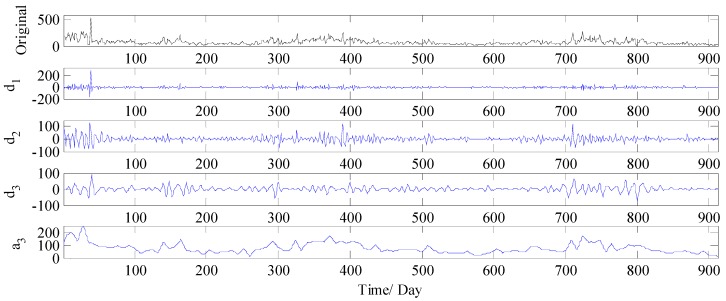
Decomposition results of PM_2.5_ concentration series by WT (Wuhan).

The multiple frequency components in PM_2.5_ concentration series are always the challenging parts in forecasting, making the models which work on the original time series cannot handle them appropriately. In order to improve the forecast accuracy, in this study, WT is firstly employed to divide the PM_2.5_ concentration series collected from Wuhan into four components including one low frequency approximation subset and three high frequency detail subsets (see Figure 8). The four components are denoted respectively as $d_{1}$, $d_{2}$, $d_{3}$ and $a_{3}$, where $a_{3}$ is the low frequency approximation subset which illustrates the general trend of the PM_2.5_ concentration series, and $d_{1}$,$d_{2}$, and $d_{3}$ indicate the high frequency detail subsets, which are related to the noise and disturbance. 

After decomposition of original PM_2.5_ concentration series by WT, DE-BP model is utilized to forecast all the subsets. There is no doubt that the PM_2.5_ concentrations of previous several days have a great influence on the latter ones. Therefore, in this study, a certain number of previous PM_2.5_ concentration data are taken as the input of DE-BP model for forecasting the latter one. After several simulations and predictions, the optimal length of predicted series is set as eight in DE-BP model in order to obtain the higher accuracy. In DE algorithm, the parameter settings are listed as follows: population size: $N_{p}=100$, scaling factor: F=0.5, crossover probability: $C_{R}=0.5$, max iterations:$G_{M}=100$. The above parameter settings and input determination method are used in all tests throughout the paper in order to ensure fair and valid comparisons between different forecasting models. 

**Figure 9:**
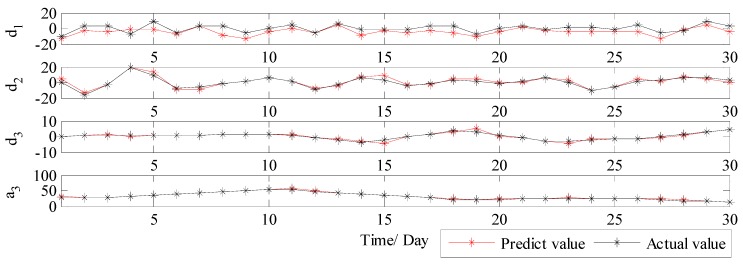
The forecast results of each subset by WT-DE-BP model (Wuhan).

Based on the above parameter settings, each subset is forecasted using DE-BP model, and the forecast results are illustrated in Figure 9. It is obvious that all the four subsets cannot be forecasted with high accuracy, especially the subsets $d_{1}$, $d_{2}$, and $d_{3}$, which are related to the noise and disturbance. Therefore, it can be concluded that the single decomposition process by WT cannot effectively extract the multiple frequency components existed in the PM_2.5_ concentration series, and therefore leads to a relatively inferior forecasting performance.

**Figure 10:**
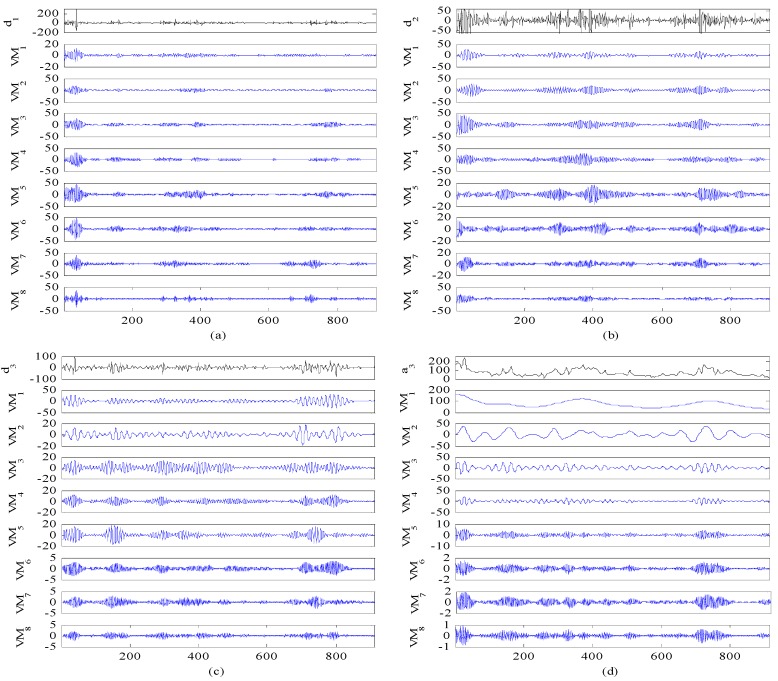
Decomposition results of each subset by VMD (Wuhan).

**Figure 11:**
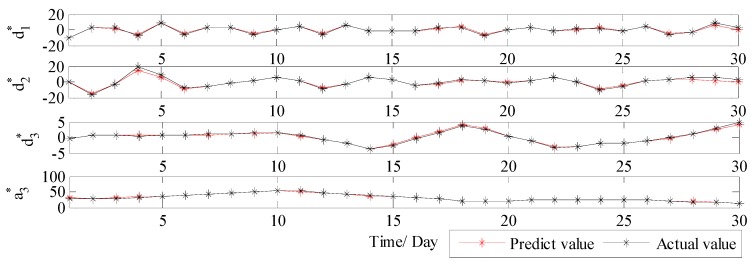
The forecast results of each subset using WT-VMD-DE-BP (Wuhan).

Therefore, in order to solve the drawback of WT and further improve the forecast accuracy, VMD is further applied to conduct the secondary decomposition of each subset generated by WT. In this study, each subset is decomposed into eight VMs, and the decomposition results of $d_{1}$, $d_{2}$, $d_{3}$ and $a_{3}$ by VMD are illustrated in Figure 10. Then, DE-BP model is employed to forecast all VMs based on the rolling technology. Next, the forecasting value of each subset is obtained by aggregation of the forecast values of all the VMs generated by VMD decomposition of this subset. Finally, the ultimate forecast result of PM_2.5_ concentration series can be obtained by adding up the forecast values of each subset. The ultimate forecast results and the corresponding MAE, RMSE, MAPE and TIC of WT-VMD-DE-BP model for all the four subsets are presented in Figure 11 and Table 1. From Figure 11 and Table 1, it is obvious that after secondary decomposition by VMD, the forecast accuracies of all the four subsets are significantly improved, which confirms the effectiveness of the hybrid decomposition technology proposed in this study.

#### 3.3.2. Results and Discussions

In this section, to verify the superiority of the proposed WT-VMD-DE-BP model in forecasting capability, forecasting models of BP, DE-BP, WT-DE-BP, VMD-DE-BP and WT-VMD-DE-BP are adopted as the benchmark models. Four error measurements, MAE, RMSE, MAPE and TIC, are employed for evaluating the performance of all the forecasting models.

**Figure 12:**
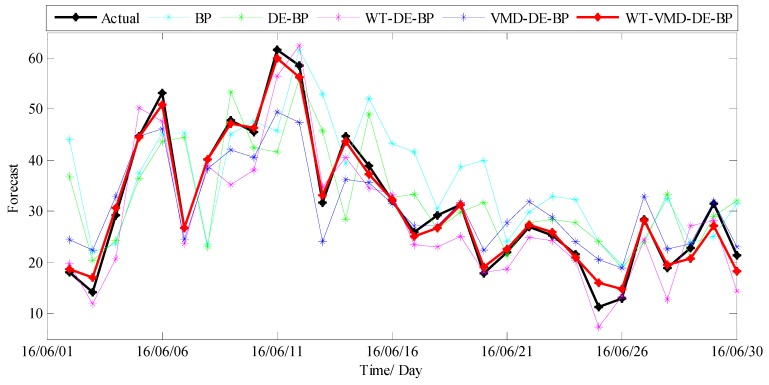
The ultimate forecast result of different models (Wuhan).

**Figure 13:**
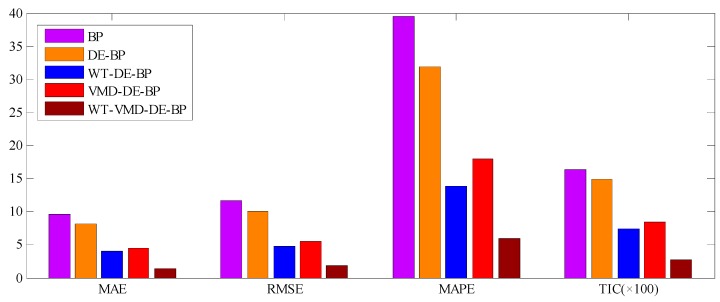
Error graphics of different models (Wuhan).

The forecast results of all considered models are shown in Figure 12, and the forecast errors including MAE, RMSE, MAPE and TIC of the proposed model and benchmark models are presented in Table 2 where the smallest value of each row is marked in boldface. As shown in Table 2, the error values of MAE, RMSE, MAPE and TIC of the proposed model are all smallest compared with all the other benchmark models, which confirms that the proposed hybrid WT-VMD-DE-BP model based on WT-VMD decomposition technique has the best forecasting performance. In order to present the comparison more intuitively, the error figures MAE, RMSE, MAPE and TIC of different models are also provided in Figure 13. 

In order to further analyze the effects of the decomposition technique and DE optimization algorithm on the proposed model, the following three categories of comparisons are conducted in this experiment. The first category of comparison (Comparison I), which is designed for testing the positive effects of single decomposition techniques, is conducted between the forecasting models embedded with single decomposition techniques (WT-DE-BP model and VMD-DE-BP model) and the forecasting models without any decomposition techniques (DE-BP model). The second category of comparison (Comparison II), which is designed for proving the advantages of hybrid WT-VMD decomposition technique, is carried out between the proposed model and the forecasting models embedded with single decomposition techniques (WT-DE-BP model and VMD-DE-BP model). The third category of comparison (Comparison III), which is designed for confirming the contribution of DE algorithm on the BP model, is conducted between the DE-BP model and BP model. The comparison results of Comparison I, Comparison II and Comparison III are all presented in Table 3. Based on the results listed in Table 3, the following three categories of findings can be obtained.

(1) Findings of Comparison I (WT-DE-BP vs. DE-BP and VMD-DE-BP vs. DE-BP)

In Table 3, it is obvious that the values of MAE, RMSE, MAPE and TIC of DE-BP model have been reduced by 50.12%, 51.08%, 56.67% and 50.00%, respectively, via integrating the WT decomposition technique into DE-BP model, and have been decreased by 44.21%, 45.04%, 44.02% and 42.86%, respectively, through combining the VMD decomposition technique into DE-BP model. Based on the above comparison results, it can be concluded that through decomposing the PM_2.5_ concentration series into a set of subsets with different frequencies, the single decomposition technique (WT and VMD) can decrease the characteristics of non-linearity and non-stability existed in the original PM_2.5_ concentration series to some extent, and thus is benefit for improving the forecasting ability of DE-BP model.

(2) Findings of Comparison II (WT-VMD-DE-BP vs. WT-DE-BP and WT-VMD-DE-BP vs. VMD-DE-BP)

In Table 2, it can be found that the values of MAE, RMSE, MAPE and TIC of WT-VMD-DE-BP model decrease by 66.91%, 62.94%, 57.01% and 57.14%, respectively, compared with those of WT-DE-BP model, and 70.42%, 67.69%, 66.72% and 62.50%, respectively, compared with those of VMD-DE-BP model. Therefore, based on the above analysis, it can be easily found that the proposed WT-VMD-DE-BP model can significantly decrease the errors including MAE, RMSE, MAPE and TIC of WT-DE-BP and VMD-DE-BP models. Thus, it can be concluded that the hybrid WT-VMD decomposition technique proposed in this paper is very effective for improving the forecast accuracy. The reason lies in that the single decomposition techniques (WT and VMD) have the drawback of mode mixing problem with different levels, which makes the multiple frequency components existed in the PM_2.5_ concentration series cannot be effectively extracted, and therefore leads to an inferior forecasting performance. 

(3) Findings of Comparison III (DE-BP vs. BP)

In Table 2, it is obvious that the values of MAE, RMSE, MAPE and TIC of BP model has been reduced by 15.50%, 13.69%, 19.14% and 14.29%, respectively, via integrating the DE algorithm into BP model. Thus, it can be concluded that through optimizing weight matrices and thresholds using DE algorithm, the BP model obtains stronger approximation ability. In addition, it can also be seen that the DE algorithm cannot effectively decrease the forecast errors without decomposition techniques (WT and VMD), which confirms that the multiple frequency components existed in the PM_2.5_ concentration series have remarkable influence on the forecast accuracy.

#### 3.3.3. PM_2.5_ Concentration Forecasting in Tianjin

**Figure 14:**
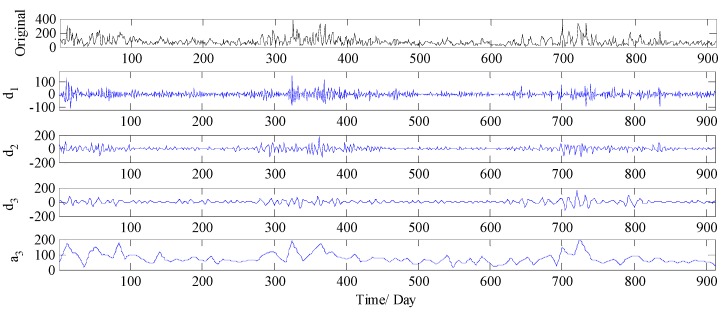
Decomposition results of PM_2.5_ concentration series by WT (Tianjin).

**Figure 15:**
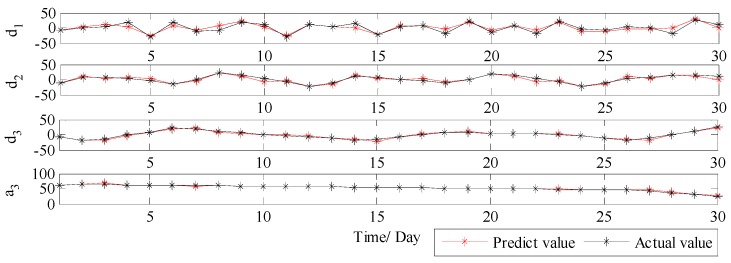
The forecast results of each subset by WT-DE-BP model (Tianjin).

**Figure 16:**
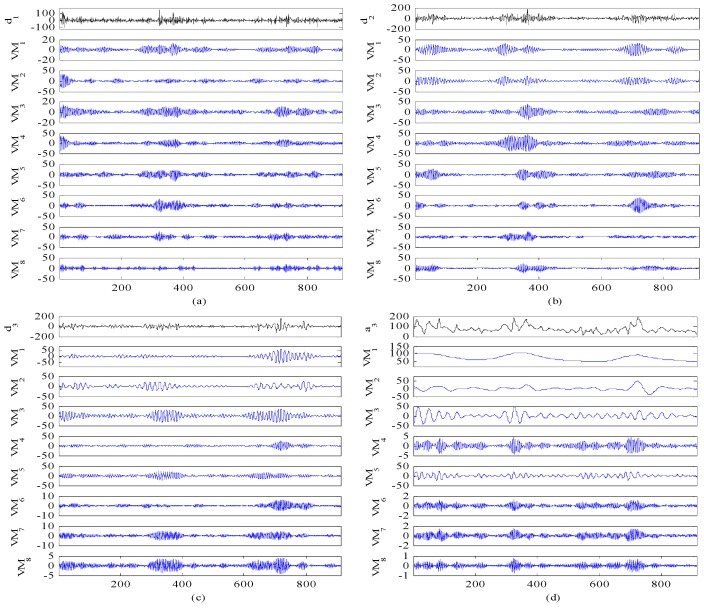
Decomposition results of each subset by VMD (Tianjin).

**Figure 17:**
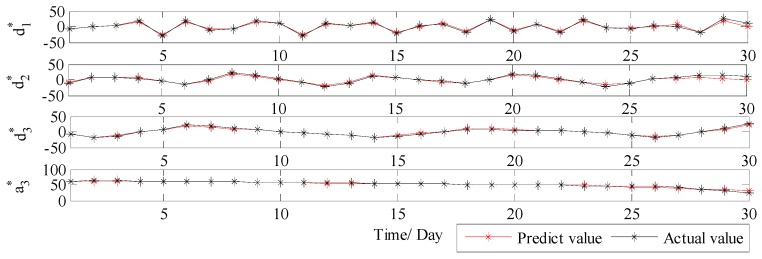
The forecast results of each subset by WT-VMD-DE-BP (Tianjin).

**Figure 18:**
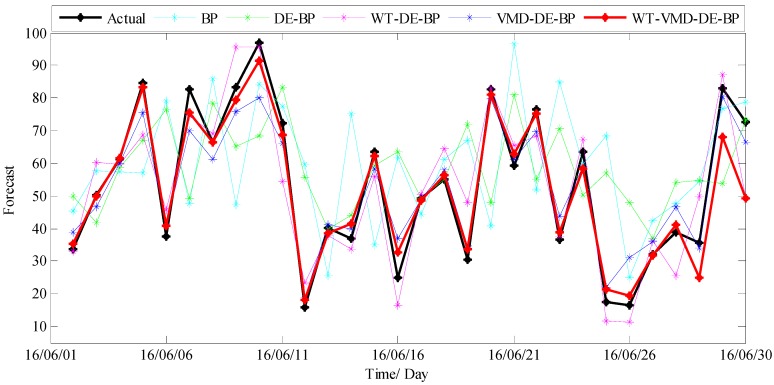
The ultimate forecast results of different models (Tianjin).

**Figure 19:**
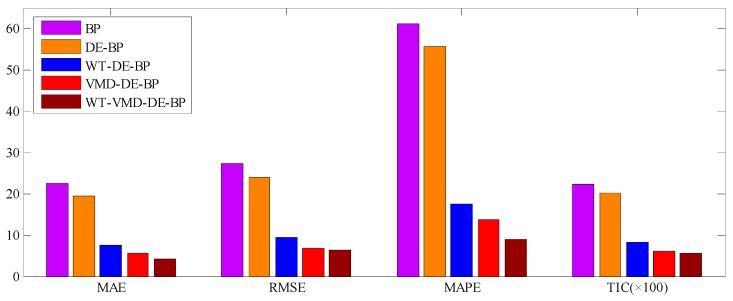
Error graphics of different models (Tianjin).

In order to further systematically and comprehensively testify the validity and applicability of the proposed WT-VMD-DE-BP model, the PM_2.5_ concentration series collected in Tianjin (see Figure 7) is also taken as another study case. Similar to the case in Wuhan, the decomposition results of original PM_2.5_ concentration series using WT decomposition method are shown in Figure 14. For each subset, the forecast result of WT-DE-BP model is shown in Figure 15. The decomposition results of each subset based on VMD decomposition method are depicted in Figure 16. In addition, for each subset, the forecast result of WT-VMD-DE-BP model is provided in Figure 17. Finally, the ultimate PM_2.5_ concentration forecast results of different models are illustrated in Figure 18. The forecast errors, MAE, RMSE, MAPE and TIC, of all the forecasting models are also calculated and displayed, respectively, in Table 4 and Table 5 and Figure 19.

Based on the simulation results, it is obvious that the similar conclusions to the case in Wuhan can be obtained. As it is shown, the proposed WT-VMD-DE-BP model owns the best performance compared with all the other considered models including BP, DE-BP, WT-DE-BP and VMD-DE-BP in this paper once again, which further confirms that the proposed model is suitable for PM_2.5_ concentration forecasting, and owns a highly application ability. Similarly, it has been demonstrated that the performance of the proposed model based on the hybrid decomposition technique (WT-VMD-DE-BP model) is much better than the models embedded with single decomposition technique (WT-DE-BP model and VMD-DE-BP model). It has also been verified that the decomposition technique (WT and VMD) can improve the forecasting ability of DE-BP model, and the DE algorithm has a positive effect on the BP model.

## 4. Conclusions

Accurate PM_2.5_ concentration forecasting is crucial for risk-analysis and decision-making in environmental protection departments. However, the multiple frequency components that exist in PM_2.5_ concentration series are always the challenging parts in forecasting, making models that work on the original time series unable to handle them appropriately. Thus, many researchers have been making efforts to solve this problem using different data decomposition techniques such as WT and VMD before forecasting. Since all single decomposition techniques have the drawback of mode mixing problem with different levels, which makes the multiple frequency components that exist in the PM_2.5_ concentration series unable to be effectively extracted, and consequently leading to an inferior forecasting performance. Therefore, in order to solve the mode mixing problem existed in the single decomposition technique, this paper, through combing the advantages of WT and VMD, proposes a novel hybrid WT-VMD decomposition technique, and then established a forecasting model based on WT-VMD and DE-BP model to improve the forecast accuracy of PM_2.5_ concentration.

In order to demonstrate the effectiveness and applicability of the proposed model, two PM_2.5_ concentration series collected from Wuhan and Tianjin located in China are taken for conducting the empirical study. Based on the experimental results, four main conclusions can be obtained as follows: (1) The proposed WT-VMD-DE-BP model owns the best performance compared with all the other considered benchmark models including BP, DE-BP, WT-DE-BP and VMD-DE-BP, which demonstrates that the proposed model is highly suitable for the non-stationary PM_2.5_ concentration forecasting; (2) The single decomposition techniques of WT and VMD cannot improve the forecasting ability of DE-BP model significantly due to the drawback of mode mixing problemwith different levels existed in WT and VMD; (3) The hybrid WT-VMD decomposition technique performs better than the single decomposition methods of WT and VMD in extracting the multiple frequency components that exist in the PM_2.5_ concentration series, thus leading to a good forecasting performance; (4) DE algorithm has a positive effect on the BP model by optimizing the weights and thresholds between input layer and hidden layer.

However, as mentioned above, the intermittent and unstable nature of PM_2.5_ concentration series makes its forecasting become a very difficult task. Therefore, there are still several research directions left for the future. For example, some meteorological factors such as atmospheric pressure, temperature, and precipitation may be integrated into the forecasting model to improve the forecast accuracy. Furthermore, since the PM_2.5_ concentration series have some similar characteristics as other time series such as non-linearity and non-stability, the proposed model in this study can also be used for other complex time series forecasting, such as forecasting of electricity load, wind speed and stock price.

## Figures and Tables

**Table 1:** Forecast errors of WT-DE-BP and WT-VMD-DE-BP for each subset.

Index	Forecast Errors of WT-DE-BP				Forecast Errors of WT-VMD-DE-BP			
	$d_{1}$	$d_{2}$	$d_{3}$	$a_{3}$	$d_{1}$	$d_{2}$	$d_{3}$	$a_{3}$
MAE	4.97	1.83	0.45	1.38	0.85	1.08	0.17	0.95
RMSE	5.79	2.31	0.75	1.89	1.05	1.58	0.21	1.20
MAPE (%)	222.38	357.01	56.56	5.42	33.75	36.04	22.83	3.80
TIC	0.52	0.17	0.18	0.03	0.12	0.13	0.05	0.02

**Table 2:** Comparison of prediction performances of different models (Wuhan).

Index	BP	DE-BP	WT-DE-BP	VMD-DE-BP	WT-VMD-DE-BP
MAE	9.61	8.12	4.05	4.53	**1.34**
RMSE	11.68	10.08	4.83	5.54	**1.79**
MAPE (%)	39.50	31.94	13.84	17.88	**5.95**
TIC	0.16	0.14	0.07	0.08	**0.03**

Note: The smallest value of each row is marked in boldface.

**Table 3:** The comparison results of Comparisons I, II and III (Wuhan).

Index	The Proportion of Reduction				
	WT-VMD-DE-BP	WT-VMD-DE-BP	WT-DE-BP	VMD-DE-BP	DE-BP
	vs.	vs.	vs.	vs.	vs.
	WT-DE-BP	VMD-DE-BP	DE-BP	DE-BP	BP
MAE (%)	66.91	70.42	50.12	44.21	15.50
RMSE (%)	62.94	67.69	51.08	45.04	13.69
MAPE (%)	57.01	66.72	56.67	44.02	19.14
TIC (%)	57.14	62.50	50.00	42.86	14.29

**Table 4:** Comparison of prediction performances of different models (Tianjin).

Index	BP	DE-BP	WT-DE-BP	VMD-DE-BP	WT-VMD-DE-BP
MAE	22.52	19.45	7.53	5.54	**4.05**
RMSE	27.28	23.81	9.50	6.79	**6.25**
MAPE(%)	61.03	55.51	17.49	13.66	**8.88**
TIC	0.22	0.20	0.08	0.06	**0.05**

Note: The smallest value of each row is marked in boldface.

**Table 5:** The comparison results of Comparisons I, II and III (Tianjin).

Index	The Proportion of Reduction				
	WT-VMD-DE-BP	WT-VMD-DE-BP	WT-DE-BP	VMD-DE-BP	DE-BP
	vs.	vs.	vs.	vs.	vs.
	WT-DE-BP	VMD-DE-BP	DE-BP	DE-BP	BP
MAE (%)	46.22	26.89	61.13	71.51	11.43
RMSE (%)	34.21	7.95	60.10	71.48	10.55
MAPE (%)	49.19	34.99	68.51	75.39	9.10
TIC (%)	37.50	16.67	60.00	70.00	9.09
